# Association of Zip Code Vaccination Rate With COVID-19 Mortality in Chicago, Illinois

**DOI:** 10.1001/jamanetworkopen.2022.14753

**Published:** 2022-05-27

**Authors:** Sharon Zeng, Kenley M. Pelzer, Robert D. Gibbons, Monica E. Peek, William F. Parker

**Affiliations:** 1 Pritzker School of Medicine, University of Chicago, Chicago, Illinois; 2 Department of Medicine, University of Chicago, Chicago, Illinois; 3 Department of Public Health Sciences, University of Chicago, Chicago, Illinois; 4 Division of the Biological Sciences, University of Chicago, Chicago, Illinois; 5 MacLean Center for Clinical Medical Ethics, University of Chicago, Chicago, Illinois

## Abstract

**Importance:**

There has been large geographic inequity in vaccination coverage across Chicago, Illinois, with higher vaccination rates in zip codes with residents who predominantly have high incomes and are White.

**Objective:**

To determine the association between inequitable zip code–level vaccination coverage and COVID-19 mortality in Chicago.

**Design, Setting, and Participants:**

This retrospective cohort study used Chicago Department of Public Health vaccination and mortality data and Cook County Medical Examiner mortality data from March 1, 2020, through November 6, 2021, to assess the association of COVID-19 mortality with zip code–level vaccination rates. Data were analyzed from June 1, 2021, to April 13, 2022.

**Exposures:**

Zip code–level first-dose vaccination rates before the Alpha and Delta waves of COVID-19.

**Main Outcomes and Measures:**

The primary outcome was deaths from COVID-19 during the Alpha and Delta waves. The association of a marginal increase in zip code–level vaccination rate with weekly mortality rates was estimated with a mixed-effects Poisson regression model, and the total number of preventable deaths in the least vaccinated quartile of zip codes was estimated with a linear difference-in-difference design.

**Results:**

The study population was 2 686 355 Chicago residents in 52 zip codes (median [IQR] age 34 [32-38] years; 1 378 658 [51%] women; 773 938 Hispanic residents [29%]; 783 916 non-Hispanic Black residents [29%]; 894 555 non-Hispanic White residents [33%]). Among residents in the least vaccinated quartile, 80% were non-Hispanic Black, compared with 8% of residents identifying as non-Hispanic Black in the most vaccinated quartile (P < .001). After controlling for age distribution and recovery from COVID-19, a 10–percentage point increase in zip code–level vaccination 6 weeks before the peak of the Alpha wave was associated with a 39% lower relative risk of death from COVID-19 (incidence rate ratio [IRR], 0.61 [95% CI, 0.52-0.72]). A 10–percentage point increase in zip code vaccination rate 6 weeks before the peak of the Delta wave was associated with a 24% lower relative risk of death (IRR, 0.76 [95% CI, 0.66-0.87]). The difference-in-difference estimate was that 119 Alpha wave deaths (72% [95% CI, 63%-81%]) and 108 Delta wave deaths (75% [95% CI, 66%-84%]) might have been prevented in the least vaccinated quartile of zip codes if it had had the vaccination coverage of the most vaccinated quartile.

**Conclusions and Relevance:**

These findings suggest that low zip code–level vaccination rates in Chicago were associated with more deaths during the Alpha and Delta waves of COVID-19 and that inequitable vaccination coverage exacerbated existing racial and ethnic disparities in COVID-19 deaths.

## Key Points

**Question:**

What was the association of vaccination coverage inequity with COVID-19 mortality in Chicago, Illinois?

**Findings:**

In this cohort study of 2 686 355 Chicago residents, higher zip code vaccination coverage was associated with lower relative risks of death during the Alpha and Delta waves of the COVID-19 pandemic. Approximately 75% of deaths in the least vaccinated zip codes may have been prevented if mortality trends had remained parallel with the most vaccinated zip codes.

**Meaning:**

These findings suggest that low zip code–level vaccination rates in Chicago were associated with more deaths in the Alpha and Delta waves, exacerbating racial and ethnic disparities in COVID-19 mortality.

## Teaser

This cohort study assesses the association between zip code–level vaccination coverage and COVID-19 mortality in Chicago, Illinois.

## Introduction

COVID-19 has killed more than 7600 people in Chicago, Illinois, as of April 2022.^1^ Structural racism, defined as differential access to goods, services, and opportunities by race, has played a substantial role in COVID-19 disparities across the country. In Chicago, Black and Hispanic residents have been disproportionately affected. By the end of the first wave in 2020, Black residents made up 43% of COVID-19 deaths despite being 29% of Chicago’s population, and Hispanic residents made up 48% of COVID-19 cases despite also being 29% of Chicago’s population.^2^ Previous studies have also found spatial correlation between the percentage of residents identifying as Black and number of COVID-19 deaths within a Chicago neighborhood.^3,4^ After the US Food and Drug Administration (FDA) granted Emergency Use Authorization for COVID-19 vaccines, a common goal of phased implementation plans across the country was to mitigate racial inequities in COVID-19 outcomes. However, predominantly White and high-income communities were often the first to receive vaccines in major cities.^5^ To our knowledge, few studies have quantified the associations of inequitable vaccine coverage with severe COVID-19 outcomes in a major US city.

COVID-19 vaccine efficacy has previously been estimated through randomized trials and multicenter studies on defined patient populations, but it is also important to empirically assess efficacy in a large and diverse population.^6,7,8,9^ Evaluating vaccine uptake and efficacy within the context of racial and ethnic residential segregation in Chicago may also inform future public health efforts in other urban settings.^10,11,12^ In this study, we quantify the association between inequitable vaccination coverage and mortality during the Alpha wave (March 28-June 19) and Delta wave (August 1-November 6) of COVID-19 in Chicago in 2021.

## Methods

### Study Design and Population

This cohort study was a secondary analysis of publicly available, deidentified data and was granted exemption status by the University of Chicago Biological Sciences Division University of Chicago Medical Center Institutional Review Board. This report follows the Strengthening the Reporting of Observational Studies in Epidemiology (STROBE) reporting guideline for cohort studies.

We used data sets from the Chicago Department of Public Health (CDPH),^13,14^ including COVID-19 infections, deaths, and vaccination coverage organized by zip code from March 1, 2020, through the week of December 19, 2021. We excluded zip codes with fewer than 10 000 residents and those that were primarily outside city limits (eTable 1 in the Supplement). For a robustness check on spatial distribution of deaths, we used Cook County Medical Examiner data with geocoded residential addresses from March 1, 2020, through December 25, 2021. Population counts and sociodemographic data were obtained from the US Census Bureau American Community Survey 5-year estimates for 2015 to 2019.^15,16^ Demographic data in the Census, including sex and race and ethnicity responses, were based on self-identification and used Census terms.

The exposure for the primary analysis was the 6-week lagged percentage of vaccinated residents in the zip code, since at least 1 dose of any FDA-approved COVID-19 vaccine provided significant protection against severe outcomes from the Alpha and Delta variants.^6,7,8,17^ We divided the zip codes into quartiles based on cumulative vaccination rate by March 28, 2021, six weeks before the peak of the Alpha wave.^18^ We calculated the population-weighted median age, sex, race and ethnicity, high school graduation rate, median household income, and health insurance rates of each vaccine group using American Community Survey data.

### Main Outcomes and Measures

The primary outcome was mortality from COVID-19, defined by the CDPH as occurring among confirmed cases with a positive molecular or antigen test result,^13^ in a given zip code during the Alpha wave (March 28 to June 19, 2021) and Delta wave (August 1 to November 6, 2021). The secondary outcome was weekly new infections in each zip code, defined by the CDPH as a positive molecular or antigen test result.^13^

### Statistical Analysis

We compared distributions of age groups, sex, and race and ethnicity across the vaccine groups using χ^2^ tests. High school graduates and health insurance were compared across all groups using analysis of variance. Median age and median household income were compared across all groups using a Kruskal-Wallis test. For all statistical tests, 2-sided *P* < .05 was considered the threshold for statistical significance.

We assessed the association of vaccination coverage with COVID-19 mortality with 3 approaches. First, we calculated an unadjusted Pearson product-moment correlation coefficient between prewave vaccination level and total wave mortality, weighted by zip code population. Using bootstrapped SEs, we ran a *t* test for significance with a null hypothesis of no correlation.

Second, to estimate the marginal outcome associated with higher vaccination rates in weekly COVID-19 death rates, we fit mixed-effects Poisson regression models using data from December 13, 2020, to June 19, 2021, for the Alpha wave and May 9, 2021, to November 6, 2021, for the Delta wave. Fixed effects in the models were weeks since the peak of the previous wave, an indicator variable for the start of the wave, and the interaction between wave and vaccination coverage 6 weeks before the peak of the wave. We constructed models for both waves and adjusted for the proportion of the population aged at least 65 years and percentage recovered from previous SARS-CoV-2 infection before the wave by including them as fixed effects. A zip code–level random intercept accounted for the clustering of weekly COVID-19 mortality rates.

Third, we performed a linear difference-in-difference analysis of cumulative COVID-19 death rates for the most and least vaccinated quartiles before and after each wave. To check the parallel trends assumption, we fit a Poisson regression model to the downslope from the December 2020 wave (December 13, 2020, to March 28, 2021) and tested the interaction between vaccination group and decline in death rate.

We performed 3 robustness checks for the Poisson regression. First, we changed the exposure to the 6-week lagged percentage of residents fully vaccinated. Next, we used vaccination group as a categorical treatment variable to assess for nonlinear effects of vaccination rate on mortality. Lastly, we used mortality data from the Cook County Medical Examiner Case Archive,^19^ which provided residential addresses that we geocoded to census tracts. Because vaccination data are collected by the CDPH and only available at the zip code level, we constructed a data set that contained weekly deaths and vaccination coverage within the geographic overlap of a census tract and zip code with population estimated from the Crosswalk Files from the US Department of Housing and Urban Development.^20^

We performed all analyses with R statistical software version 4.0.5 (R Project for Statistical Computing). We have posted data sets and code online.^21^ Data were analyzed from June 1, 2021, to April 13, 2022.

## Results

### Study Population

A total of 2 686 355 Chicago residents in 52 zip codes were included in the analysis, with 773 938 residents (29%) identifying as Hispanic, 175 220 residents (7%) identifying as non-Hispanic Asian, 783 916 residents (29%) identifying as non-Hispanic Black, and 894 555 residents (33%) identifying as non-Hispanic White (eTable 2 in the Supplement). The median (IQR) age was 34 (32-38) years. Approximately 90% of the civilian noninstitutionalized population (2 659 714 residents) had health insurance, and the median (IQR) household income was $52 044 ($41 158-$92 595). There were 6125 COVID-19 deaths recorded by the CDPH during our study period.

### Vaccination Rate and Demographics by Zip Code

At the start of the Alpha wave, the first-dose vaccination rate ranged from 18% to 27% in the least vaccinated quartile (13 zip codes; population 619 518), 28% to 39% in the intermediate quartiles (26 zip codes; population 1 582 146), and 40% to 49% in the most vaccinated quartile (13 zip codes; population 484 691) (eTable 3 in the Supplement). While vaccine coverage across all zip codes increased to 37% to 80% by the Delta wave, the relative distribution of coverage was unchanged (eFigure in the Supplement) except for 2 zip codes in the most vaccinated quartile.

In the least vaccinated quartile, 92 994 residents (15%) were aged at least 65 years, compared with 55 670 residents (11%) in the most vaccinated quartile and 185 599 residents (12%) in the intermediate quartiles (*P* < .001). In the least vaccinated quartile, 498 263 residents (80%) identified as non-Hispanic Black, compared with 39 005 residents (8%) in the most vaccinated quartile and 246 648 residents (16%) in the intermediate quartiles (*P* < .001). In the intermediate quartiles, 638 721 residents (40%) identified as Hispanic, compared with 54 243 (11%) in the most vaccinated quartile and 80 974 residents (13%) in the least vaccinated quartile (*P* < .001). In the most vaccinated quartile, 327 806 residents (68%) identified as non-Hispanic White, compared with 538 835 residents (34%) in the intermediate quartiles and 27 914 residents (5%) in the least vaccinated quartile (*P* < .001) (Table 1).

**Table 1.** Demographics of Zip Codes by Vaccination Rate

Characteristic	Residents, No. (%)			*P* value^a^
	Least vaccinated quartile (n = 619 518)	Middle 25%-75% (n = 1 582 146)	Most vaccinated quartile (n = 484 691)	
Zip code population with ≥1 dose by March 28, 2021, %	22	33	43	NA
Age, y^b^				
0-17	151 766 (24)	350 272 (22)	59 282 (12)	<.001
18-64	374 758 (60)	1 046 275 (66)	369 739 (76)	
≥65	92 994 (15)	185 599 (12)	55 670 (11)	
Median (IQR)	36 (34-40)	34 (33-38)	33 (32-37)	.26
Sex				
Women	337 097 (54)	797 669 (50)	243 892 (50)	<.001
Men	282 421 (46)	784 477 (50)	240 799 (50)	
Race/ethnicity				
Hispanic or Latino	80 974 (13)	638 721 (40)	54 243 (11)	<.001
Non-Hispanic or Latino				
Asian	4103 (1)	121 594 (8)	49 523 (10)	
Black	498 263 (80)	246 648 (16)	39 005 (8)	
White	27 914 (5)	538 835 (34)	327 806 (68)	
Other^c^	8264 (1)	36 348 (2)	14 114 (3)	
≥High school graduate^d^	336 956 (83)	883 791 (82)	361 664 (95)	<.001
With health insurance^e^	557 180 (91)	1 385 479 (89)	459 730 (96)	<.001
Household income, median (IQR), $	34 535 (26 900-38 955)	53 864 (45 335-70 547)	94 859 (93 989-111 438)	.04

Abbreviation: NA, not appliable.

^a^ χ^2^ tests were used to compare distributions of age (*df* = 4), sex (*df* = 2), and race and ethnicity (*df* = 8) across all 3 groups. High school graduates and health insurance were compared across all groups using analysis of variance (*df* = 2). Median age and household income were compared across all groups using a Kruskal-Wallis test (*df* = 2).

^b^ Total number across the group (percentage of group population).

^c^ May include individuals identifying as American Indian or Alaska Native.

^d^ Percentages are calculated among group population older than 25 years. Least vaccinated quartile, 405 693 residents; middle quartiles, 1 074 983 residents; and most vaccinated quartile, 378 753 residents.

^e^ Percentages are calculated among group civilian noninstitutionalized population. Least vaccinated quartile, 615 196 residents; middle quartiles, 1 564 004 residents; and most vaccinated quartile, 480 514 residents.

In the most vaccinated quartile, 459 730 noninstitutionalized civilian residents (96%) had health insurance coverage, compared with 557 180 residents (91%) in the least vaccinated quartile and 1 385 479 residents (89%) in the intermediate quartiles (*P* < .001). The median (IQR) household income in the most vaccinated quartile was $94 589 ($93 989-$111 438), compared with $34 535 ($26 900-$38 955) in the least vaccinated quartile and $53 864 ($45 335-$70 547) in the intermediate quartiles (*P* = .04).

### Association of Vaccination Coverage With COVID-19 Mortality

**Figure 1.**
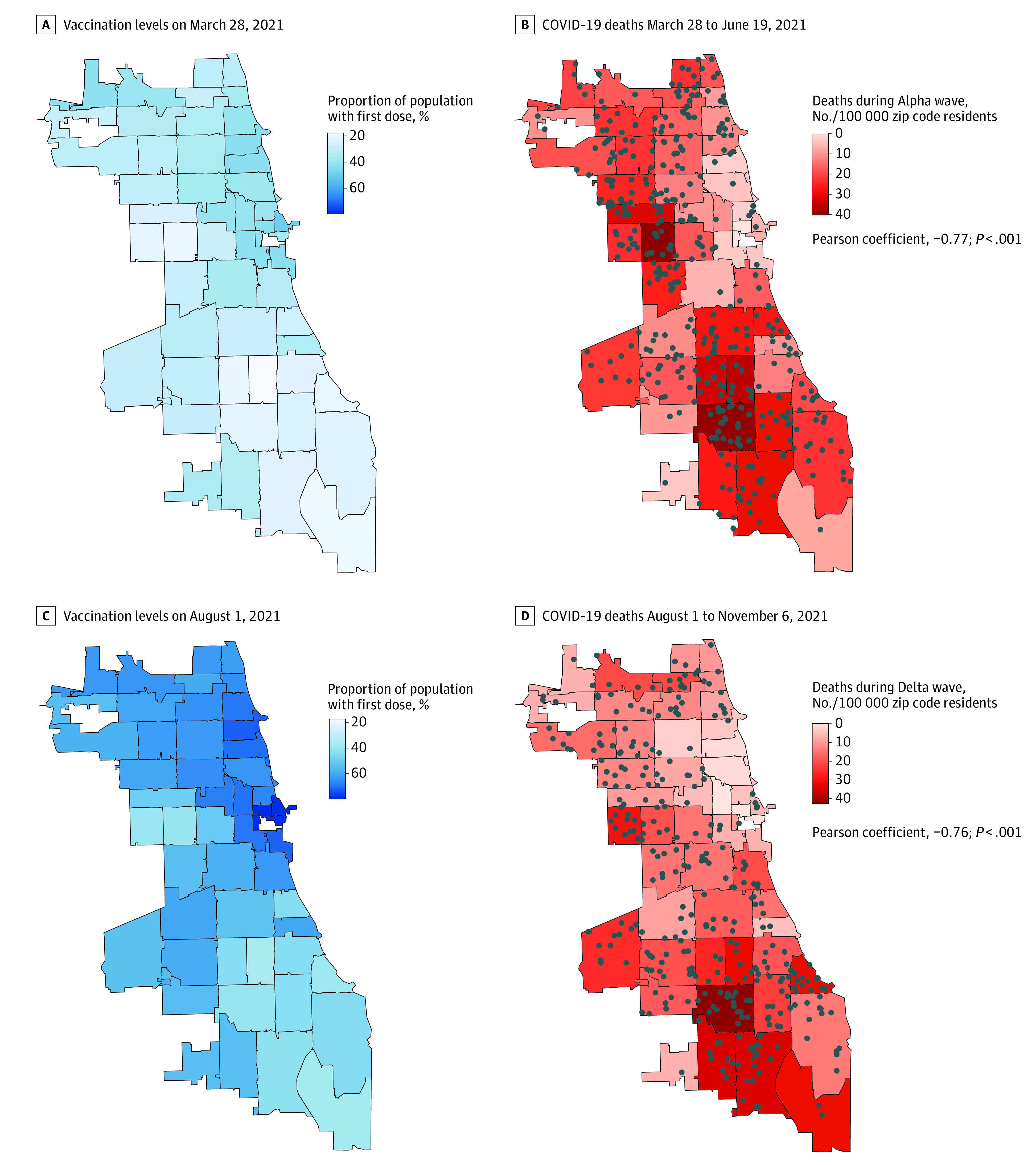
Geographic Distribution of Prewave Vaccination Coverage and Mortality Individual deaths recorded by Cook County Medical Examiner are shown in dots. Unadjusted Pearson product-moment correlation coefficient between prewave vaccination coverage and total mortality was weighted by zip code population. The *P* value was obtained from a *t* test for significance with a null hypothesis of no correlation (*r* = 0) and bootstrapped SEs.

The population-weighted Pearson correlation between vaccination level and COVID-19 deaths per 100 000 population was *r* = −0.77 (*P* < .001) during the Alpha wave and *r* = −0.76 (*P* < .001) during the Delta wave (Figure 1). A 10–percentage point increase in residents with at least 1 dose 6 weeks prior to the Alpha wave peak was associated with a 39% decrease in the weekly risk of death from COVID-19 (incidence rate ratio [IRR], 0.61 [95% CI, 0.51-0.72]), as estimated by the mixed-effects Poisson regression model. After adjusting for the percentage of residents aged at least 65 years and the percentage of residents who had recovered from SARS-CoV-2 infection (Table 2; eTable 4 in the Supplement), the risk was virtually identical (IRR, 0.61 [95% CI, 0.52-0.72]). The model estimated that increasing vaccination coverage from 20% to 45% in the least vaccinated quartile before the Alpha wave would have reduced the absolute wave mortality rate from 24.4 to 7.1 deaths per 100 000 population (absolute difference, 17.3 [95% CI, 14.1-19.1] deaths).

**Table 2.** Multivariable Analysis of Vaccination Coverage and Associated Risk Reduction in COVID-19 Deaths

Wave	Factor	IRR (95% CI)^a^	*P* value	aIRR (95% CI)^a^	*P* value
Alpha	Week	0.90 (0.88-0.91)	<.001	0.90 (0.88-0.91)	<.001
	Wave	11.1 (6.47-19.1)	<.001	10.9 (1.64-2.70)	<.001
	Wave × vaccination coverage^b^	0.61 (0.51-0.72)	<.001	0.61 (0.52-0.72)	<.001
Delta	Week	0.94 (0.92-0.96)	<.001	0.94 (0.92-0.96)	<.001
	Wave	13.8 (5.88-32.3)	<.001	13.8 (6.05-31.3)	<.001
	Wave × vaccination coverage^b^	0.76 (0.65-0.88)	<.001	0.76 (0.66-0.87)	<.001

Abbreviations: aIRR, adjusted incidence rate ratio; IRR, incidence rate ratio.

^a^ Estimates of IRRs were obtained from a mixed-effects Poisson generalized linear model with log link using fixed effects (wave and the association between wave and vaccination coverage) and random effects of zip code on intercept. aIRRs were estimated from a second model which included percentage of population older than 65 years and percentage of population previously recovered from COVID-19 as fixed effects. The IRR for week was calculated for a 1-week change. The wave IRR was calculated with an indicator variable for after the beginning of each wave (March 28, 2021, for Alpha and August 1, 2021, for Delta). CIs and *P* values were obtained from asymptotic Wald tests. Full model output is reported in eTable 4 (Alpha wave) and eTable 5 (Delta wave) in the Supplement.

^b^ For vaccination coverage, a 1-unit increase corresponds to a 10–percentage point increase in people with at least 1 dose.

During the Delta wave, a 10–percentage point increase in vaccination rate 6 weeks prior to the Delta wave peak was associated with a 24% decrease in weekly risk of death from COVID-19 (IRR, 0.76 [95% CI, 0.65-0.88]). After adjusting for residents aged at least 65 years and previous SARS-CoV-2 infections, a 10–percentage point increase was associated with a virtually identical risk (IRR, 0.76 [95% CI, 0.66-0.87) (Table 2; eTable 5 in the Supplement). The model estimated that increasing vaccination coverage from 40% to 70% in the least vaccinated quartile 6 weeks prior to the Delta wave would reduce the Delta wave death rate from 19 deaths per 100 000 residents to 8.2 deaths per 100 000 residents (absolute difference, 10.8 [95% CI, 7.7-11.1] deaths).

### Difference-in-Difference Estimate of Vaccine-Preventable Deaths

**Figure 2.**
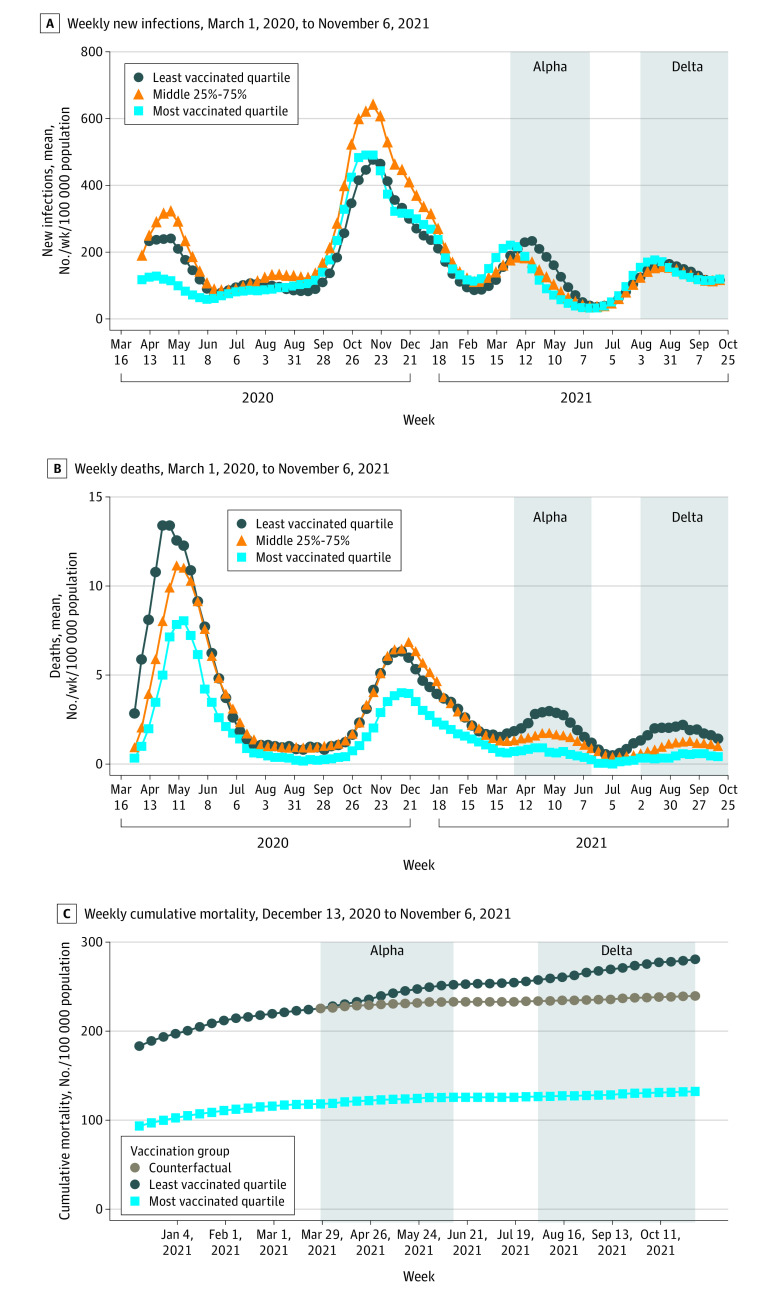
COVID-19 Outcomes by Vaccination Group Centers of the rolling means are plotted. C, Brown circles indicate the counterfactual scenario in which the least vaccinated group experienced the same trend in cumulative mortality as the most vaccinated group starting on March 28, 2021.

During the Alpha wave, there were 36 deaths in the most vaccinated quartile, 165 deaths in the least vaccinated quartile, and 46 deaths in the counterfactual scenario for the least vaccinated quartile. The cumulative COVID-19 mortality increased from 225.5 to 252.1 deaths per 100 000 residents in the least vaccinated quartile (26.6 additional deaths per 100 000 residents), compared with 118.2 to 125.6 deaths per 100 000 residents in the most vaccinated quartile (7.4 additional deaths per 100 000), a difference-in-difference of 19.2 deaths per 100 000 residents. The difference-in-difference estimate for deaths that may have been prevented was 119 (95% CI, 104-134) deaths per 100 000 residents, corresponding to 72% (95% CI, 63%-81%) of all Alpha wave deaths in the least vaccinated quartile (Figure 2C).

During the Delta wave, there were 28 deaths in the most vaccinated quartile, 144 deaths in the least vaccinated quartile, and 36 deaths in the counterfactual scenario for the least vaccinated quartile. The cumulative COVID-19 mortality increased from 257.5 to 280.7 deaths per 100 000 residents in the least vaccinated quartile (23.2 additional deaths per 100 000 residents) compared with 126.5 to 132.2 deaths per 100 000 residents in the most vaccinated quartile (5.7 additional deaths per 100 000 residents), a difference-in-difference of 17.4 deaths per 100 000 residents. The difference-in-difference estimate for deaths that may have been prevented was 108 (95% CI, 95-121) deaths per 100 000 residents, corresponding to 75% (95% CI, 66%-84%) of all Delta wave deaths in the least vaccinated group. A likelihood ratio test of Poisson regression analysis failed to reject the null hypothesis of parallel trends in weekly mortality between vaccination groups during the decline of the pre-Alpha wave of deaths (eTable 12 in the Supplement).

Across both waves, there were a total of 342 deaths in the least vaccinated quartile, compared with 68 deaths in the most vaccinated quartile. In the counterfactual scenario, 87 people would have died in the least vaccinated group. Thus, the difference-in-difference estimates that 255 (95% CI, 234-276) deaths might have been avoidable across the Alpha and Delta waves if the least vaccinated quartile had continued to follow the same trend in cumulative deaths as the most vaccinated quartile. Overall, the percentage of deaths that might have been averted was 75% (95% CI, 68%-81%).

### Mixed-Effects Poisson Model Sensitivity Analyses

A 10–percentage point increase in fully vaccinated residents was associated with decreased risk of mortality during the Alpha wave (IRR, 0.45 [95% CI, 0.34-0.59]) and the Delta wave (IRR, 0.76 [95% CI, 0.66-0.87]) (eTable 6 in the Supplement). The lowest vaccination quartile was associated with greater weekly risk of death from COVID-19 compared with the highest vaccination quartile during the Alpha wave (IRR, 2.53 [95% CI, 1.71-3.75]), and during the Delta wave (IRR, 2.25 [95% CI, 1.35-3.75]) (eTable 7 in the Supplement). Using 2-week lags, there was decreased risk for the Alpha wave (IRR, 0.72 [95% CI, 0.64-0.80]) and for the Delta wave (IRR, 0.75 [95% CI, 0.65-0.87]) (eTable 8 in the Supplement). Using 8-week lags also resulted in decreased risk for the Alpha wave (IRR, 0.48 [95% CI, 0.38-0.62]) and the Delta wave (IRR, 0.76 [95% CI, 0.66-0.87) (eTable 9 in the Supplement). Finally, when using census tract–zip code overlap as a more granular geographic unit, the risk was still decreased for the Alpha wave (IRR, 0.61 [95% CI, 0.51-0.72]) (eTable 10 in the Supplement) and the Delta wave (IRR, 0.68 [95% CI, 0.61-0.77) (eTable 11 in the Supplement).

## Discussion

This cohort study of Chicago COVID-19 mortality leverages detailed geospatial data to estimate the deadly associations of inequity in vaccination coverage. While the burden of severe COVID-19 outcomes was always unequally distributed across zip codes, prior to the vaccination campaign, there existed consistent parallel trends in pandemic mortality between the different parts of the city. During the Alpha wave, a large gap in COVID-19 death rate opened between the most and least vaccinated zip codes and continued to widen throughout the Delta wave. If the least vaccinated quartile had continued to follow the same trend as the most vaccinated quartile, an estimated 75% of COVID-19 deaths may have been averted in these hardest-hit areas. In a mixed-effects Poisson regression model, a 10–percentage point increase in vaccination rate was associated with a 39% reduction in deaths during the Alpha wave and a 24% reduction during the Delta wave. The least vaccinated zip codes had more residents who identified as Black or Hispanic and more low-income and uninsured residents, suggesting that structural racism contributed to inequities in vaccine coverage.

Our more granular zip code–level findings are consistent with the state-level association between vaccination rate and subsequent COVID-19 mortality.^9^ Although the gap in mortality between vaccination groups widened during the Alpha and Delta waves, the trends in weekly new infections remained parallel. This discrepancy could indicate that first-dose vaccination levels during our study period were not high enough to sustain herd immunity against infection, but more likely reflects structural inequity in access to testing and surveillance practices.^22,23,24^ These large systematic disparities in case detection rate across Chicago would severely bias any attempt to estimate the association of vaccine coverage on infections.

Following the approach of the Center for Medicare & Medicaid Services in evaluating hospital performance,^25^ we did not condition our analyses on socioeconomic factors, such as race and ethnicity, income, education, or insurance status. Adjusting for these factors in a regression model could obscure the geographic disparity in vaccine-preventable deaths and minimize the impact of poor health care delivery to disadvantaged populations.

Further research is necessary to directly examine the mechanism of inequitable vaccination coverage. Our study focused on the Alpha and Delta waves of COVID-19 deaths in Chicago, 2 time periods of the pandemic in which different factors may have led to inequitable coverage. Prior to the Alpha wave, vaccine doses were extremely scarce and distribution was heavily dependent on allocation policy decisions. Although large academic hospitals had the resources to vaccinate more people, they were often unreachable for Black and Hispanic communities.^26^ Early scheduling methods favored those with English fluency and the resources to make appointments as soon as web pages refreshed, and food and agricultural workers who had disproportionately less flexibility to take time off work to get vaccinated.^27,28^ When Chicago put in place a temporary program to vaccinate communities that had experienced disproportionately severe COVID-19 outcomes, the racial and ethnic gap in coverage decreased, suggesting that problems of access, as opposed to vaccine hesitancy, were the primary contributors to unequal vaccination coverage in early phases of vaccine availability.^29^ However, by the beginning of the Delta wave, vaccines were more available in Chicago, yet vaccination coverage remained low in marginalized communities. Studies have shown that individuals from marginalized racial and ethnic groups in Chicago are more hesitant about COVID-19 vaccines compared with White counterparts.^30,31^ Structural racism in health care has also been cited as one of the factors driving vaccine hesitancy and medical distrust in the US, particularly among individuals who identify as Black.^32,33^

Whether through barriers to access, medical distrust, or other factors influencing the decision to pursue vaccination, vaccination efforts in Chicago failed to provide equitable coverage to Black and Hispanic communities. This inequality in vaccination coverage is likely to have played a major role in exacerbating the racial and ethnic disparity in deaths during the Alpha and Delta waves of COVID-19.

### Limitations

Our study has several limitations. First, we had zip code–level (not patient-level) data on vaccination coverage, which limited our ability to control for observed confounders. Second, unmeasured time-varying zip code–level confounders, such as changing distributions in age owing to COVID-19 mortality, could bias our difference-in-difference estimates. However, parallel trends between the high and low vaccination quartiles during the pre-Alpha wave make confounding by time-invariant unobserved variables unlikely. Third, zip code–level differences in prevalence of comorbid conditions, as well as ability to socially distance and therefore exposure to infection, affect how much of the disparity in deaths can be attributed to differences in vaccination coverage. Additionally, the data integrity depends on the accurate reporting of the residence. Individuals experiencing homelessness or those without a permanent address are unlikely to be accounted for accurately.

## Conclusions

This cohort study found that prior to the Alpha wave of COVID-19 in Chicago, the highest vaccination rates were disproportionately in zip codes with high incomes and predominantly White populations. During the Alpha and Delta waves, highly vaccinated areas broke with historical mortality trends and had substantially fewer deaths. Inequitable vaccination coverage in Chicago exacerbated existing racial and ethnic disparities in COVID-19 mortality.

## Notes

Supplement. **eFigure.** Visualizations of Alpha Vaccination Groups **eTable 1.** Zip Codes Excluded From Analysis **eTable 2.** Demographics of Study Population **eTable 3.** List of Zip Codes and Groups Included in Analysis **eTable 4.** Alpha Wave Complete Mixed-Effects Models Output **eTable 5.** Delta Wave Complete Mixed-Effects Models Output **eTable 6.** Sensitivity Analyses Using Percentage of Population Fully Vaccinated **eTable 7.** Sensitivity Analysis Using Vaccination Groups **eTable 8.** Sensitivity Analysis With 2-Week Lag Period **eTable 9.** Sensitivity Analysis With 8-Week Lag Period **eTable 10.** Sensitivity Analysis Using Census Tract-Level Data (Alpha Wave) **eTable 11.** Sensitivity Analysis Using Census Tract-Level Data (Delta Wave) **eTable 12.** Test for Parallel Trends
