# Supplementary material for: Association of Zip Code Vaccination Rate With COVID-19 Mortality in Chicago, Illinois
Source: JAMA Netw Open. 2022 May 27;5(5):e2214753. doi: 10.1001/jamanetworkopen.2022.14753 (PMC9142872; doi:10.1001/jamanetworkopen.2022.14753)
Supplement: Supplement. — eFigure. Visualizations of Alpha Vaccination Groups eTable 1. Zip Codes Excluded From Analysis eTable 2. Demographics of Study Population eTable 3. List of Zip Codes and Groups Included in Analysis eTable 4. Alpha Wave Complete Mixed-Effects Models Output eTable 5. Delta Wave Complete Mixed-Effects Models Output eTable 6. Sensitivity Analyses Using Percentage of Population Fully Vaccinated eTable 7. Sensitivity Analysis Using Vaccination Groups eTable 8. Sensitivity Analysis With 2-Week Lag Period eTable 9. Sensitivity Analysis With 8-Week Lag Period eTable 10. Sensitivity Analysis Using Census Tract-Level Data (Alpha Wave) eTable 11. Sensitivity Analysis Using Census Tract-Level Data (Delta Wave) eTable 12. Test for Parallel Trends [file jamanetwopen-e2214753-s001.pdf]

Supplemental Online Content

Zeng S, Pelzer KM, Gibbons RD, Peek ME, Parker WF. Association of zip code vaccination rate with COVID-19 mortality in Chicago, Illinois. *JAMA Netw Open*. 2022;5(5):e2214753. doi:10.1001/jamanetworkopen.2022.14753

eFigure. Visualizations of Alpha Vaccination Groups
eTable 1. Zip Codes Excluded From Analysis
eTable 2. Demographics of Study Population
eTable 3. List of Zip Codes and Groups Included in Analysis
eTable 4. Alpha Wave Complete Mixed-Effects Models Output
eTable 5. Delta Wave Complete Mixed-Effects Models Output
eTable 6. Sensitivity Analyses Using Percentage of Population Fully Vaccinated
eTable 7. Sensitivity Analysis Using Vaccination Groups
eTable 8. Sensitivity Analysis With 2-Week Lag Period
eTable 9. Sensitivity Analysis With 8-Week Lag Period
eTable 10. Sensitivity Analysis Using Census Tract-Level Data (Alpha Wave)
eTable 11. Sensitivity Analysis Using Census Tract-Level Data (Delta Wave)
eTable 12. Test for Parallel Trends

This supplemental material has been provided by the authors to give readers additional information about their work.

© 2022 Zeng S et al. *JAMA Network Open.*

eFigure. Visualizations of Alpha Vaccination Groups

(a) Line graph showing cumulative vaccinations in each ZIP code from December 13, 2020, to November 6, 2021. Color and dot shape represent vaccination groups to which the ZIP codes were assigned. Vertical lines denote the first date of vaccination phases in Chicago (Phase 1A: residential healthcare facilities and healthcare workers; 4 weeks after Phase 1A: Estimated date that the earliest vaccine effect would be detectable; Phase 1B: aged 65+, non-healthcare residential settings, and frontline essential workers; Phase 1C: aged 16-64 with underlying medical conditions and all other essential workers; Phase 2: remaining Chicagoans aged 16+). **(b)** Histogram of vaccination levels. **(c)** Map of ZIP codes.

(a)

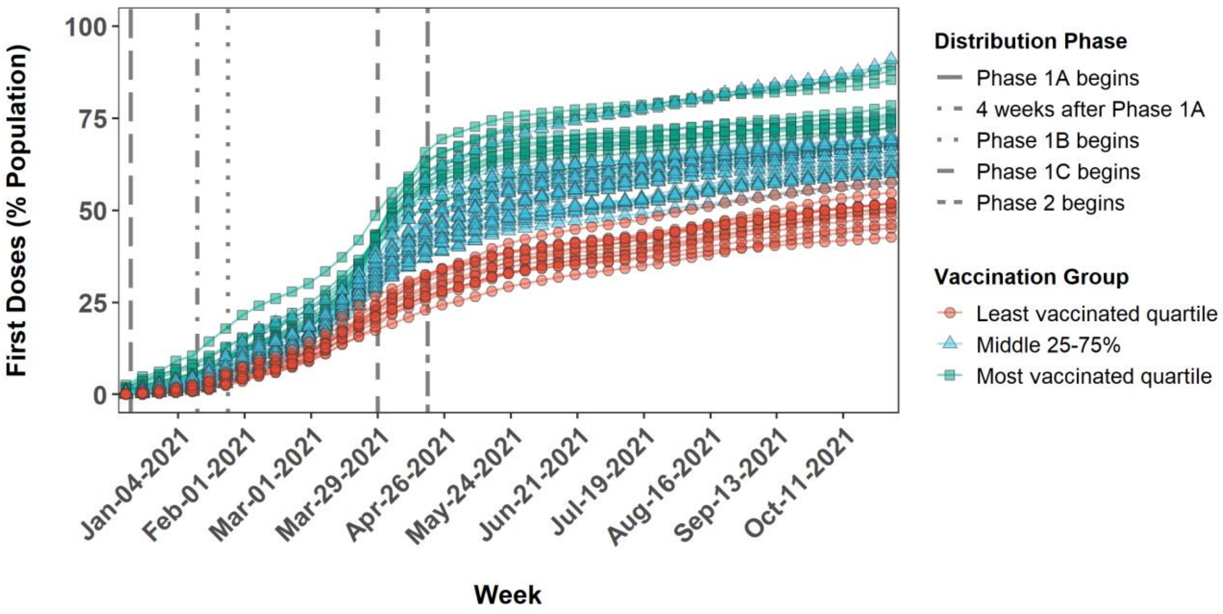

© 2022 Zeng S et al. *JAMA Network Open.*

(b)

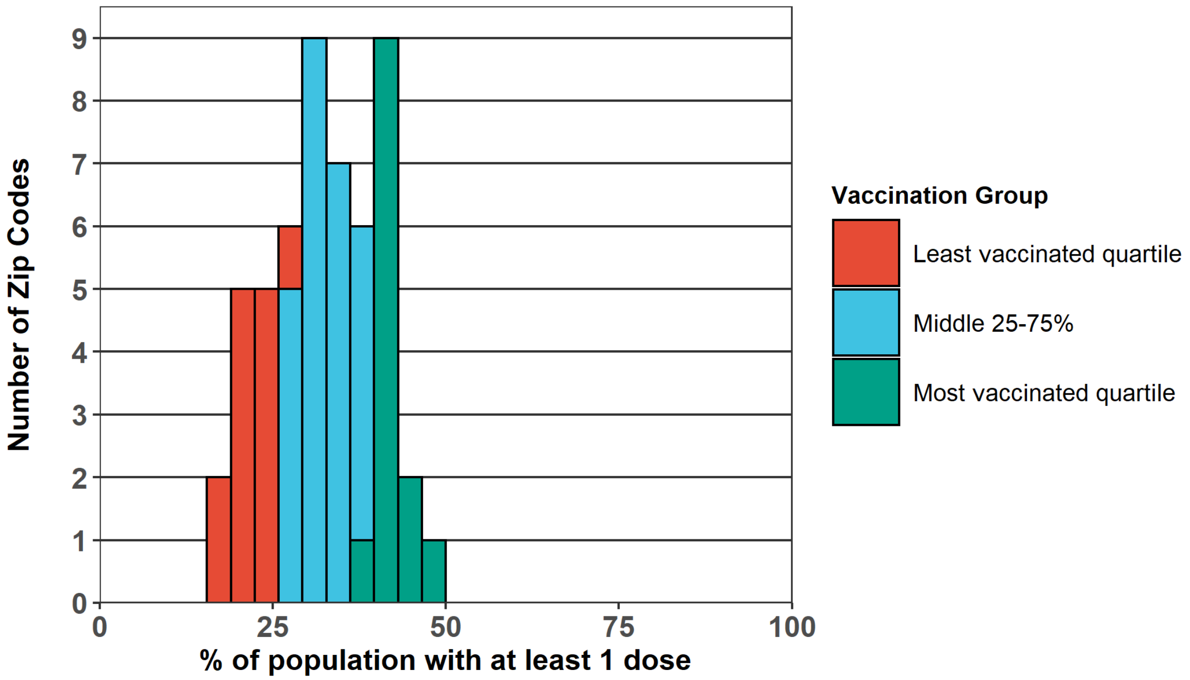

© 2022 Zeng S et al. *JAMA Network Open.*

(c)

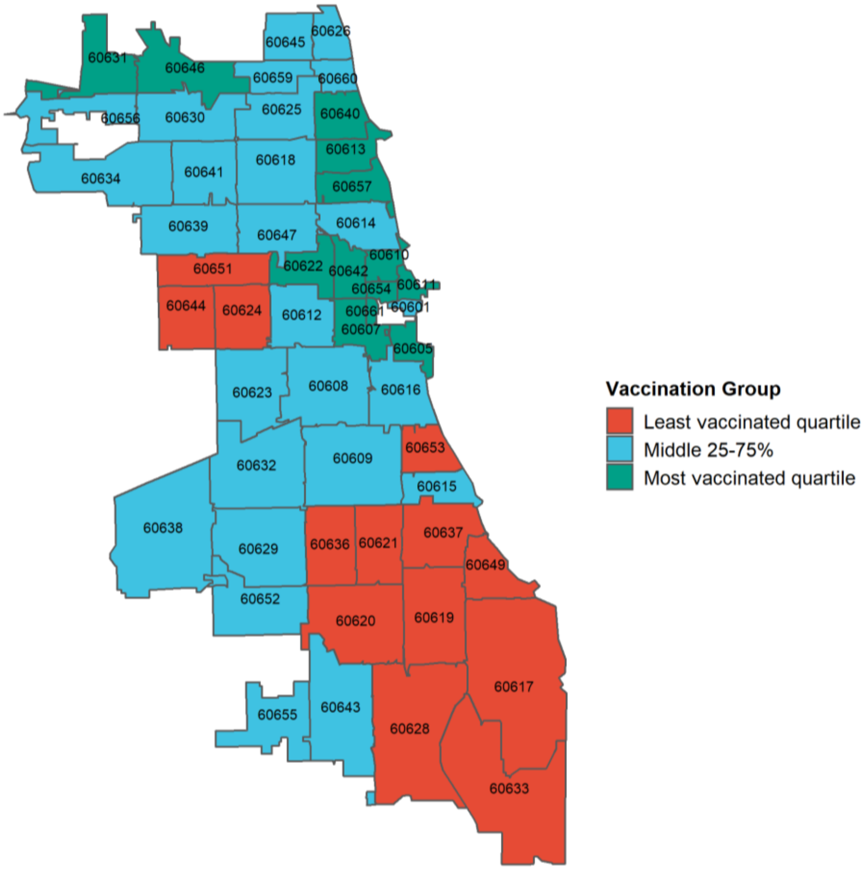

© 2022 Zeng S et al. *JAMA Network Open.*

eTable 1. Zip Codes Excluded From Analysis

List of ZIP codes excluded from analyses. Five ZIP codes (60602, 60603, 60604, 60606, 60666) were excluded because they contained fewer than 10,000 residents. 60707 and 60827 were excluded because they lie mostly outside the City of Chicago limits.

ZIP	Vaccination Group	Number of First Doses by 3/28/21	% First Doses by 3/28/21	Number of First Doses by 8/1/21	% First Doses by 8/1/21	Population
**60602**	Outliers	683	59.7	1281	112	1145
**60603**	Outliers	682	64.8	1465	139	1052
**60604**	Outliers	389	47.3	772	93.8	823
**60606**	Outliers	1570	47.8	3165	96.3	3287
**60666**	Outliers	55	0	92	0	0
**60707**	Outliers	4830				
**60827**	Outliers	451				

© 2022 Zeng S et al. *JAMA Network Open.*

eTable 2. Demographics of Study Population

**Population**	2686355
**Age**	**No. (%)[a]**
Age 0-17	561320 (21)
Age 18-65	1790772 (67)
Age 65+	334263 (12)
**Median Age Years (IQR)**	34 (32-38)
**Sex**	**No. (%)**
Female	1378658 (51)
Male	1307697 (49)
**Race and Ethnicity**	**No. (%)**
Hispanic or Latino	773938 (29)
Non-Hispanic or Latino	
Asian	175220 (7)
Black	783916 (29)
White	894555 (33)
Other	58726 (2)
**High School Graduate or Higher No. (%)[b]**	1582411 (84)
**With Health Insurance No. (%)[c]**	2402389 (90)
**Median Household Income $ (IQR)**	52044 (41158-92595)

[a] No. (%) = total number (percentage of total population).
[b] Calculated as percentage of population over 25 years old (1,859,429).
[c] Calculated as percentage of civilian noninstitutionalized population (2,659,714).

© 2022 Zeng S et al. *JAMA Network Open.*

eTable 3. List of Zip Codes and Groups Included in Analysis

ZIP	Vaccination Group	Number of First Doses by 3/28/21	% First Doses by 3/28/21	Number of First Doses by 8/1/21	% First Doses by 8/1/21	Population
**60621**	Least vaccinated quartile	4913	17.5	10202	36.4	28018
**60633**	Least vaccinated quartile	2348	18.5	4789	37.7	12689
**60624**	Least vaccinated quartile	6791	19.5	14168	40.6	34892
**60636**	Least vaccinated quartile	6156	20.5	13279	44.2	30024
**60649**	Least vaccinated quartile	9845	21.1	18082	38.8	46633
**60644**	Least vaccinated quartile	9881	21.2	18694	40.1	46591
**60620**	Least vaccinated quartile	15043	22.2	27911	41.2	67711
**60637**	Least vaccinated quartile	10682	22.6	20881	44.1	47300
**60628**	Least vaccinated quartile	14684	22.9	27199	42.3	64254
**60617**	Least vaccinated quartile	19283	23.1	37536	44.9	83553
**60651**	Least vaccinated quartile	15366	24.2	31428	49.5	63492
**60619**	Least vaccinated quartile	14962	24.4	26729	43.7	61207
**60653**	Least vaccinated quartile	8846	26.7	14680	44.3	33154
**60659**	Middle 25-75%	11879	27.8	25582	59.9	42735
**60612**	Middle 25-75%	9636	28.6	17032	50.5	33735
**60609**	Middle 25-75%	17628	28.9	32543	53.4	60939
**60656**	Middle 25-75%	8219	29.1	15244	54	28218
**60623**	Middle 25-75%	23703	29.2	44275	54.5	81283
**60638**	Middle 25-75%	17467	29.8	31360	53.5	58669
**60652**	Middle 25-75%	13147	30.3	24083	55.4	43447
**60639**	Middle 25-75%	26810	30.4	53431	60.6	88204
**60629**	Middle 25-75%	33615	30.6	65216	59.3	110029
**60634**	Middle 25-75%	23311	31	43882	58.4	75082
**60641**	Middle 25-75%	21998	31.5	43708	62.5	69880
**60645**	Middle 25-75%	14982	31.7	29784	63	47270

© 2022 Zeng S et al. *JAMA Network Open.*

**60626**	Middle 25-75%	16552	32.7	31519	62.4	50544
**60632**	Middle 25-75%	29379	32.7	54483	60.6	89857
**60616**	Middle 25-75%	18074	33.3	34301	63.3	54197
**60630**	Middle 25-75%	18824	33.4	34332	60.8	56433
**60643**	Middle 25-75%	16370	33.5	26497	54.2	48887
**60615**	Middle 25-75%	13978	34.4	24558	60.5	40590
**60625**	Middle 25-75%	27379	34.5	51326	64.6	79444
**60618**	Middle 25-75%	33329	35.1	59896	63.1	94907
**60655**	Middle 25-75%	10130	35.5	15553	54.4	28569
**60608**	Middle 25-75%	29498	36.8	46767	58.4	80059
**60647**	Middle 25-75%	32408	37	57271	65.4	87633
**60660**	Middle 25-75%	16444	37	28895	64.9	44498
**60601**	Middle 25-75%	5765	38.2	11937	79.1	15083
**60614**	Middle 25-75%	28064	39	46283	64.3	71954
**60610**	Most vaccinated quartile	16074	39.6	27005	66.6	40548
**60646**	Most vaccinated quartile	11482	40.2	18259	63.9	28569
**60622**	Most vaccinated quartile	21756	40.8	36258	68	53294
**60640**	Most vaccinated quartile	28646	41.3	47968	69.2	69363
**60642**	Most vaccinated quartile	8140	41.3	13835	70.2	19716
**60631**	Most vaccinated quartile	12378	41.9	18709	63.4	29529
**60607**	Most vaccinated quartile	12377	42.3	20350	69.5	29293
**60661**	Most vaccinated quartile	4413	42.6	8253	79.7	10354
**60605**	Most vaccinated quartile	12467	42.9	20925	72	29060
**60657**	Most vaccinated quartile	30412	42.9	50052	70.5	70958
**60654**	Most vaccinated quartile	8678	43.3	15959	79.7	20022
**60613**	Most vaccinated quartile	22026	43.4	36686	72.3	50761
**60611**	Most vaccinated quartile	16135	48.6	26188	78.8	33224

© 2022 Zeng S et al. *JAMA Network Open.*

eTable 4. Alpha Wave Complete Mixed-Effects Models Output

To analyze the correlation between vaccination levels on March 28, 2021, and alpha wave-specific COVID-19 mortality, we narrowed our dataset to start at December 13, 2020, the peak of deaths during the second wave of COVID-19 mortality, and analyzed data from this date to the end of the alpha wave on June 20, 2021. We fit the data to the following models:

(1) $$ln(Y_i) - ln(Population) = \beta_{0i} + \beta_1(Week) + \beta_2(Wave) + \beta_3(Wave \times Vaccination\ Coverage)$$

(2) $$ln(Y_i) - ln(Population) = \beta_{0i} + \beta_1(Week) + \beta_2(Wave) + \beta_3(Wave \times Vaccination\ Coverage) + \beta_4(Recovered) + \beta_5(65+)$$

Where $\beta_{0i} = \beta_0 + v_{0i}$ and is a randomly distributed intercept with random effects structure

$$v_{o_i} \sim N(0, \sigma_v^2)$$

$\boldsymbol{Week}$ is a continuous variable describing the number of weeks since the start of the data, $Wave$ is a categorical variable describing whether at date was in the second or alpha wave, $\boldsymbol{Recovered}$ is a continuous variable describing the percentage of ZIP code residents who had recovered from COVID-19 by March 28, 2021, and $65+$ is a continuous variable describing the percentage of ZIP code residents who are at least 65 years old.

$\boldsymbol{Wave}$ is a categorical indicator variable for date after March 28th, 2021.

$\boldsymbol{Wave} \times \boldsymbol{Vaccination\ Coverage}$ describes the effect of a 10-percentage point increase in vaccinations by March 28, 2021, on the number of deaths per week in the alpha wave.

© 2022 Zeng S et al. *JAMA Network Open.*

eTable 4: Alpha wave complete mixed-effects models output (continued)

	Unadjusted			Adjusted		
**Predictors**	**Estimate**	**Std. Error**	**p**	**Estimate**	**Std. Error**	**p**
(Intercept)	-9.87	0.08	<0.001	-11.56	0.28	<0.001
Week	-0.11	0.01	<0.001	-0.11	0.01	<0.001
Wave	2.41	0.28	<0.001	2.39	0.27	<0.001
Wave×Vaccination Coverage	-0.50	0.09	<0.001	-0.49	0.08	<0.001
Recovered				0.84	0.19	<0.001
65+				0.74	0.13	<0.001
**Random Effects**						
σ^2	0.74			0.74		
τ_{00}	0.18 zip			0.07 zip		
ICC	0.19			0.08		
N	52 zip			52 zip		
Observations	1404			1404		
Marginal R^2 / Conditional R^2	0.292 / 0.430			0.394 / 0.445		

© 2022 Zeng S et al. *JAMA Network Open.*

eTable 5. Delta Wave Complete Mixed-Effects Models Output

The same model structure described in equations 1 and 2 (see above in **eTable 4**) was also used to estimate the association between vaccination levels on August 1, 2021, and delta wave-specific COVID-19 mortality. We analyzed data from the peak of the alpha wave of deaths on May 9, 2021, to the end of the delta wave on November 6, 2021.

$\boldsymbol{Week}$ is a continuous variable describing the number of weeks since the start of the data,

$\boldsymbol{Wave}$ is a categorical variable describing whether if the date was after August 1, 2021

$\boldsymbol{Recovered}$ is a continuous variable describing the percentage of ZIP code residents who had recovered from COVID-19 by August 1, 2021, and $65+$ is a continuous variable describing the percentage of ZIP code residents who are at least 65 years old.

$\boldsymbol{Wave} \times \boldsymbol{Vaccination\ Coverage}$ describes the effect of a 10-percentage point increase in vaccinations by August 1, 2021, on the number of deaths per week in the delta wave.

	Unadjusted				Adjusted		
**Predictors**	**Estimate**	**Std. Error**	**p**		**Estimate**	**Std. Error**	**p**
(Intercept)	-11.42	0.10	<0.001		-12.87	0.39	<0.001
Week	-0.07	0.01	<0.001		-0.07	0.01	<0.001
Wave	2.62	0.43	<0.001		2.62	0.42	<0.001
Wave×Vaccination Coverage	-0.28	0.09	<0.001		-0.28	0.07	<0.001
Recovered					0.51	0.51	0.039
65+					0.73	0.17	<0.001
**Random Effects**							
σ^2	1.31				1.31		
τ_{00}	0.17 $_{zip}$				0.09 $_{zip}$		
ICC	0.12				0.06		
N	52 $_{zip}$				52 $_{zip}$		
Observations	1352				1352		
Marginal R^2 / Conditional R^2	0.073 / 0.182				0.154 / 0.208		

© 2022 Zeng S et al. *JAMA Network Open.*

eTable 6. Sensitivity Analyses Using Percentage of Population Fully Vaccinated

We ran Model 2 using the percentage of residents fully vaccinated by March 28, 2021 (alpha wave) or August 1, 2021 (delta wave), rather than the percentage of residents with at least one dose. A 1 unit increase of $\boldsymbol{Wave} \times \boldsymbol{Fully\ Vaccinated\ Coverage}$ corresponds to a 10-percentage point increase in fully vaccinated residents.

	Alpha				Delta		
**Predictors**	**Estimate**	**IRR (CI)**	**p**		**Estimate**	**IRR (CI)**	**p**
(Intercept)	-11.64	0.00 (0.00 – 0.00)	<0.001		-12.86	0.00 (0.00 – 0.00)	<0.001
Week	-0.11	0.90 (0.88 – 0.91)	<0.001		-0.07	0.94 (0.92 – 0.96)	<0.001
Wave	2.26	9.55 (5.81 – 15.70)	<0.001		2.47	11.77 (5.50 – 25.17)	<0.001
Recovered	0.85	2.34 (1.61 – 3.42)	<0.001		0.50	1.64 (1.02 – 2.66)	0.043
65+	0.80	2.22 (1.73 – 2.85)	<0.001		0.74	2.09 (1.50 – 2.91)	<0.001
Wave×Fully Vaccinated Coverage	-0.80	0.45 (0.34 – 0.59)	<0.001		-0.27	0.76 (0.66 – 0.87)	<0.001
**Random Effects**							
σ^2	0.74				1.31		
$\tau_{00\ zip}$	0.07 $_{zip}$				0.09 $_{zip}$		
ICC	0.09				0.06		
N $_{zip}$	52 $_{zip}$				52 $_{zip}$		
Observations	1404				1352		
Marginal R^2/ Conditional R^2	0.386/ 0.437				0.153/ 0.206		

© 2022 Zeng S et al. *JAMA Network Open.*

eTable 7. Sensitivity Analysis Using Vaccination Groups

As another sensitivity analysis, we ran Model 2 using the categorical variable $\boldsymbol{Week} \times \boldsymbol{Vaccination\_Group}$ rather than continuous variable $\boldsymbol{Wave} \times \boldsymbol{Vaccinations}$. The reference group was $[\boldsymbol{Most\ vaccinated\ quartile}]$.

For the delta wave analysis, we re-assigned ZIP codes to quartiles based on first-dose vaccination levels at beginning of the delta wave of deaths on August 1, 2021. All of the ZIP codes which were in the least vaccinated quartile before the alpha wave remained in this quartile before the delta wave. Two of the ZIP codes in the most vaccinated quartile from the alpha wave were swapped with ZIP codes from the middle 25-75%.

	Alpha				Delta		
**Predictors**	**Estimate**	**IRR (CI)**	**p**		**Estimate**	**IRR (CI)**	**p**
(Intercept)	-11.66	0.00<br>(0.00 – 0.00)	<0.001		-12.85	0.00<br>(0.00 – 0.00)	<0.001
Week	-0.11	0.90<br>(0.88 – 0.91)	<0.001		-0.07	0.94<br>(0.92 – 0.96)	<0.001
Wave	0.42	1.53<br>(1.03 – 2.27)	0.034		0.53	1.69<br>(1.00 – 2.86)	0.048
Recovered	0.92	2.51<br>(1.69 – 3.73)	<0.001		0.47	1.60<br>(0.93 – 2.76)	0.089
65+	0.76	2.14<br>(1.65 – 2.77)	<0.001		0.74	2.09<br>(1.46 – 3.00)	<0.001
Wave×Vaccination_Group<br>[Least vaccinated quartile]	0.92	2.53<br>(1.71 – 3.75)	<0.001		0.81	2.25<br>(1.35 – 3.75)	0.001
Wave×Vaccination_Group<br>[Middle 25-75%]	0.33	1.42<br>(0.97 – 2.07)	0.089		0.54	1.71<br>(1.07 – 2.74)	0.026
**Random Effects**							
σ^2	0.75				1.31		
$\tau_{00\ zip}$	0.08 $_{zip}$				0.12 $_{zip}$		
ICC	0.09				0.08		
N_{zip}	52 $_{zip}$				52 $_{zip}$		
Observations	1404				1352		
Marginal R^2/<br>Conditional R^2	0.381/<br>0.437				0.149/<br>0.220		

© 2022 Zeng S et al. *JAMA Network Open.*

eTable 8. Sensitivity Analysis With 2-Week Lag Period

In addition to the Poisson regression model using a 6-week lag period as outlined in the main text, we ran Model 2 (see page 10) using the percentage of residents fully vaccinated by 2 weeks before the peaks of the alpha and delta waves (April 25, 2021, and August 29, 2021, respectively).

	Alpha				Delta		
**Predictors**	**Estimate**	**IRR (CI)**	**p**		**Estimate**	**IRR (CI)**	**p**
(Intercept)	-11.57	0.00 (0.00 – 0.00)	<0.001		-12.90	0.00 (0.00 – 0.00)	<0.001
Week	-0.11	0.90 (0.88 – 0.91)	<0.001		-0.07	0.94 (0.92 – 0.96)	<0.001
Wave	2.32	10.17 (6.11 – 16.94)	<0.001		2.74	15.54 (6.45 – 37.40)	<0.001
Recovered 2 Weeks	0.83	2.30 (1.58 – 3.35)	<0.001		0.51	1.67 (1.03 – 2.72)	0.04
65+	0.72	2.05 (1.60 – 2.64)	<0.001		0.74	2.09 (1.49 – 2.92)	<0.001
Wave×Vaccination Coverage 2 Weeks	-0.33	0.72 (0.64 – 0.80)	<0.001		-0.29	0.75 (0.65 – 0.87)	<0.001
**Random Effects**							
σ^2	0.74				1.31		
$\tau_{00\ zip}$	0.07 $_{zip}$				0.09 $_{zip}$		
ICC	0.09				0.07		
N $_{zip}$	52 $_{zip}$				52 $_{zip}$		
Observations	1404				1352		
Marginal R^2/ Conditional R^2	0.401/ 0.452				0.154/ 0.209		

© 2022 Zeng S et al. *JAMA Network Open.*

eTable 9. Sensitivity Analysis With 8-Week Lag Period

In addition to the Poisson regression model using a 6-week lag period as outlined in the main text, we ran Model 2 using the percentage of residents fully vaccinated by 8 weeks before the peaks of the alpha and delta waves (March 14, 2021, and July 18, 2021, respectively).

	Alpha				Delta		
**Predictors**	**Estimate**	**IRR (CI)**	**p**		**Estimate**	**IRR (CI)**	**p**
(Intercept)	-11.61	0.00<br>(0.00 – 0.00)	<0.001		-12.85	0.00<br>(0.00 – 0.00)	<0.001
Week	-0.11	0.90<br>(0.88 – 0.91)	<0.001		-0.07	0.94<br>(0.92 – 0.96)	<0.001
Wave	2.51	12.30<br>(6.98 – 21.69)	<0.001		2.57	13.08<br>(5.90 – 29.02)	<0.001
Recovered 2 Weeks	0.88	2.41<br>(1.67 – 3.50)	<0.001		0.50	1.65<br>(1.02 – 2.66)	0.04
65+	0.78	2.17<br>(1.70 – 2.78)	<0.001		0.73	2.08<br>(1.49 – 2.90)	<0.001
Wave×Vaccination Coverage 2 Weeks	-0.73	0.48<br>(0.38 – 0.62)	<0.001		-0.28	0.76<br>(0.66 – 0.87)	<0.001
**Random Effects**							
σ^2	0.74				1.31		
τ_{00} zip	0.07 zip				0.09 zip		
ICC	0.08				0.06		
N zip	52 zip				52 zip		
Observations	1404				1352		
Marginal R^2/<br>Conditional R^2	0.386/<br>0.436				0.156/<br>0.208		

© 2022 Zeng S et al. *JAMA Network Open.*

eTable 10. Sensitivity Analysis Using Census Tract-Level Data (Alpha Wave)

As the final sensitivity analysis, we ran Models 1 and 2 using data from the Cook County Medical Examiner's office, which provided more granular spatial data through geocoded addresses of incidence for COVID-19 deaths in Chicago. For infectious diseases, address of incidence is usually the deceased's place of residence. We manually verified that hospitals were not being reported as address of incidence and used these coordinates to find the census tract of each death and summed the number of deaths that occurred across a census tract each week. Because vaccination data is recorded by the CDPH and only available at the ZIP code level, we mapped census tracts onto zip codes such that each observation in the dataset comprised the weekly number of deaths that occurred within the geographic overlap of a census tract and ZIP code ("overlap group"). After excluding the 7 ZIP codes listed in **eTable 2**, there were 1137 of these overlap groups in the dataset.

Out of the deaths recorded by the Medical Examiner from the peak of the second wave on December 13, 2020, to the end of the alpha wave on June 19, 2021, there were 1,409 with geocoded addresses, compared to 1,721 deaths recorded in the CDPH dataset during the same time period.

© 2022 Zeng S et al. *JAMA Network Open.*

eTable 10: Sensitivity analysis using census tract-level data (alpha wave) (continued)

	Unadjusted			Adjusted		
**Predictors**	**Estimate**	**Std. Error**	**p**	**Estimate**	**Std. Error**	**p**
(Intercept)	-10.15	0.06	<0.001	-11.69	0.23	<0.001
Week	-0.11	0.01	<0.001	-0.11	0.01	<0.001
Wave	2.67	0.28	<0.001	2.46	0.29	<0.001
Wave×Vaccination Coverage	-0.57	0.09	<0.001	-0.50	0.09	<0.001
Recovered				0.83	0.14	<0.001
65+				0.62	0.11	<0.001
**Random Effects**						
σ^2	3.70			3.70		
τ_{00}	0.37 overlap_group			0.35 overlap_group		
ICC	0.10			0.09		
N	1137 overlap_group			1137 overlap_group		
Observations	30699			30699		
Marginal R^2 / Conditional R^2	0.081 / 0.169			0.102 / 0.179		

© 2022 Zeng S et al. *JAMA Network Open.*

eTable 11. Sensitivity Analysis Using Census Tract-Level Data (Delta Wave)

We repeated the analysis from Models 1 and 2 on census tract and ZIP code overlap groups for the delta wave, using data from the peak of the alpha wave on May 9, 2021, to the end of the delta wave on November 6, 2021. There were 565 deaths with geocoded addresses from this time period in the Medical Examiner data, compared to 677 deaths recorded in the CDPH dataset.

	Unadjusted				Adjusted		
**Predictors**	**Estimate**	**Std. Error**	**p**		**Estimate**	**Std. Error**	**p**
(Intercept)	-11.67	0.10	<0.001		-13.19	0.34	<0.001
Week	-0.07	0.01	<0.001		-0.07	0.01	<0.001
Wave	3.76	0.37	<0.001		3.30	0.39	<0.001
Wave×Vaccination Coverage	-0.46	0.06	<0.001		-0.38	0.06	<0.001
Recovered					0.54	0.54	0.006
65+					0.75	0.15	<0.001
**Random Effects**							
σ^2	4.52				4.52		
τ_{00}	0.36 overlap_group				0.36 overlap_group		
ICC	0.07				0.07		
N	1137 overlap_group				1137 overlap_group		
Observations	29562				29562		
Marginal R^2 / Conditional R^2	0.038 / 0.109				0.058 / 0.124		

© 2022 Zeng S et al. *JAMA Network Open.*

eTable 12. Test for Parallel Trends

To test the parallel trends assumption during the decline in second wave COVID-19 deaths, we fit the following model to the ZIP code-level data from December 13, 2020 to the week of March 28, 2021:

(3) $$\ln(Y_i) - \ln(Population) = \beta_0 + \beta_1(Week) + \beta_2(Vaccination\_Group) + \beta_3(Week \times Vaccination\_Group)$$

Where $\boldsymbol{Week \times Vaccination\_Group}$ describes the effect of $\boldsymbol{Vaccination\_Group}$ on weekly slope during the decline of the second wave, with $[\boldsymbol{Most\ vaccinated\ quartile}]$ as the reference group.

A likelihood ratio test for $\boldsymbol{Week \times Vaccination\_Group}_{[\boldsymbol{Least\ vaccinated\ quartile}]} = \boldsymbol{Week \times Vaccination\_Group}_{[\boldsymbol{Middle\ 25-75\%}]} = \boldsymbol{0}$ with 2 degrees of freedom resulted in a Chi-squared probability of 1.825 with a p-value of 0.4015. Thus, we fail to reject the null hypothesis that there was not a significant association of $Vaccination\_Group$ on the weekly slope prior to the alpha wave of COVID-19 deaths.

**Predictors**	**Estimate**	**Std. Error**	**p**
(Intercept)	-10.15	0.13	<0.001
Week	-0.13	0.02	<0.001
Vaccination_Group [Least vaccinated quartile]	0.44	0.16	0.007
Vaccination_Group [Middle 25-75%]	0.59	0.14	<0.001
Week * Vaccination_Group [Least vaccinated quartile]	0.02	0.02	0.412
Week * Vaccination_Group [Middle 25-75%]	-0.00	0.02	0.961
Observations	832		
R^2 Nagelkerke	0.488		

© 2022 Zeng S et al. *JAMA Network Open.*
